# MAP kinase-interacting serine/threonine kinase 2 promotes proliferation, metastasis, and predicts poor prognosis in non-small cell lung cancer

**DOI:** 10.1038/s41598-017-10397-9

**Published:** 2017-09-06

**Authors:** Zhihua Guo, Guilin Peng, Ermao Li, Shaoyan Xi, Yu Zhang, Yong Li, Xiaodong Lin, Guangqiu Li, Qinian Wu, Jianxing He

**Affiliations:** 1 Department of Thoracic Surgery, The First Affiliated Hospital of Guangzhou Medical University, Guangzhou, China; 2 Guangzhou Research Institute of Respiratory Disease, Guangzhou, China; 3 Key cite of National Clinical Research Center for Respiratory Disease, Guangzhou, China; 4 Department of Urology Surgery, The First Affiliated Hospital of Guangzhou Medical University, Guangzhou, China; 5 Department of Pathology, Sun Yat-sen University Cancer Center, Guangzhou, China; 6 Department of Pathology, The First Affiliated Hospital of Guangzhou Medical University, Guangzhou, China

## Abstract

We hypothesized that MAP kinase-interacting serine/threonine kinase 2 (MNK2) may contribute to non-small cell lung cancer (NSCLC) development, and serve as a new therapeutic target. Immunohistochemical staining evaluated the correlation between MNK2 expression and clinicopathological features in 367 NSCLC cancer tissues. We determined the effects of MNK2 silencing in NSCLC cell lines *in vitro* and *in vivo*. RT-PCR and western blotting was used to examine the impact of MNK2 on ERK and AKT pathways. MNK2 was overexpressed in NSCLC cell lines and tumor tissues. Patients with MNK2 overexpression had lower OS rates (*P* < 0.001). High expression of MNK2 was correlated with lymph node metastasis (*P* = 0.008). MNK2 functioned as an independent prognostic factor for poor survival in patients with NSCLC (*P* = 0.003). MNK2 down-regulation inhibited proliferation, migration and invasion *in vitro* (*P* < 0.001), and reduced tumor growth and invasion in nude mice (*P* < 0.05). MNK2 enhanced phosphorylation of eIF4E, a downstream target of ERK and AKT pathways, which promoted NSCLC proliferation and invasion. We conclude that MNK2 overexpression in NSCLC is associated with proliferation, migration, invasion, and lower survival rates in patients via the phosphorylated eIF4E-mediated signaling pathway.

## Introduction

Lung cancer has been the leading cause of cancer mortality and morbidity globally, especially in China, where the epidemic has been established more recently^1^. The 5-year survival rates of lung cancer patients correlate with mediastinal lymph node metastasis. Although surgical resection is the optimal treatment for resecttable non-small cell lung cancer (NSCLC), overall survival (OS) is still unsatisfactory^2^. In order to improve OS and disease-free survival (DFS), adjuvant chemotherapy is generally indicated for patients with resected stages IIA through IIIA NSCLC, and radiation therapy in the treatment of unresectable lung cancer. However, clinical trials have shown that combination regimens consisting of 3 cytotoxic drugs produce greater toxicity without improving outcomes in lung cancer. Targeted therapy is a very popular treatment mode in recent years since the introduction of Gefitinib (TKI) in 2003^3^, and an increasing number of targeted therapy drugs including Bevacizumab and Crizotinib have been applied in clinical therapies. Despite the remarkable progress of targeted therapy, drug resistance can occur through the mitogen-activated protein kinase (MAPK) pathway^4^.

MAP kinase-interacting serine/threonine kinase (MNK) has been identified as a subfamily of murine serine/threonine kinase by screening a mouse embryo library with a novel extracellular signal-regulated kinase (ERK)-interacting clone as probe. MNK can bind to phosphorylated eukaryotic translation initiation factor 4E (eIF4E) to mediate early gene transcription, leading to cell proliferation or differentiation through the ERK-MAPK pathway^5, 6^. Phosphorylated eIF4E (p-eIF4E) is overexpressed in a broad spectrum of human cancers, including cancer of lung, gastric, prostate, colorectal, breast, and penile, when compared with matched adjacent normal tissues^7–11^. Indeed, eIF4E is highly phosphorylated in these tumors and correlates with poor survival and tumor progression, particularly in the early stage of tumorigenesis^7–9, 11^. Serine 209 of eIF4E is the best known phosphorylation site of MNKs^10–14^. The effect of eIF4E on tumor has been investigated by studying the mechanisms of MNKs. It was reported that MNK1/2 phosphorylation of eIF4E on serine 209 involves anti-apoptotic activity enhancement, and eIF4E’s oncogenic action promotes tumorigenesis, tumor development, and cell transformation^9, 10, 12, 13, 15, 16^. MNK1/2 deficiency presents a non-detectable level of eIF4E phosphorylation, delays tumor development, and decreases *in vitro* oncogenic activity of glioma cells^17^. Negative feedback of MNK/eIF4E pathway results in generation of anti-apoptotic, pro-survival signals^18^. MNK2 knockout mice show no eIF4E phosphorylation and have significantly attenuated tumor growth^16, 19^. P-MNK1 and p-eIF4E were elevated in nasopharyngeal carcinoma, astrocytoma, and ovarian cancer, predicting poor prognosis^20–22^. But there were less report that MNK2 has been associated with tumor prognosis. Because of MNK1/2 kinases are dispensable for cell growth and embryogenesis, and MNK2 has higher constitutive ability to phosphorylate eIF4E^5^. We propose that MNK2 may be an attractive anti-cancer target and likely to have minimal side effects.

Herein, we tested MNK2 in NSCLC to observe the role of MNK2 for malignant development and progression. In our study, overexpression of MNK2 was frequently detected in primary NSCLC cases, which correlated with lymph node metastasis, and poor OS. Functional studies found that MNK2 increased cell motility, migration and invasion *in vitro*, and promoted tumor proliferation and metastasis *in vivo*. MEK and AKT Inhibitors PD 0325901 and BEZ 235, treated NSCLC cells to show that MNK2 and its response genes were downregulated in mRNA and protein levels. Silencing MNK2 resulted in decreased phosphorylation of eIF4E and 4EBP1. In NSCLC tissues, the expression of p-eIF4E was positively correlated with MNK2 in NSCLC tissues.

## Results

### Frequent overexpression of MNK2 in NSCLC

**Figure 1:**
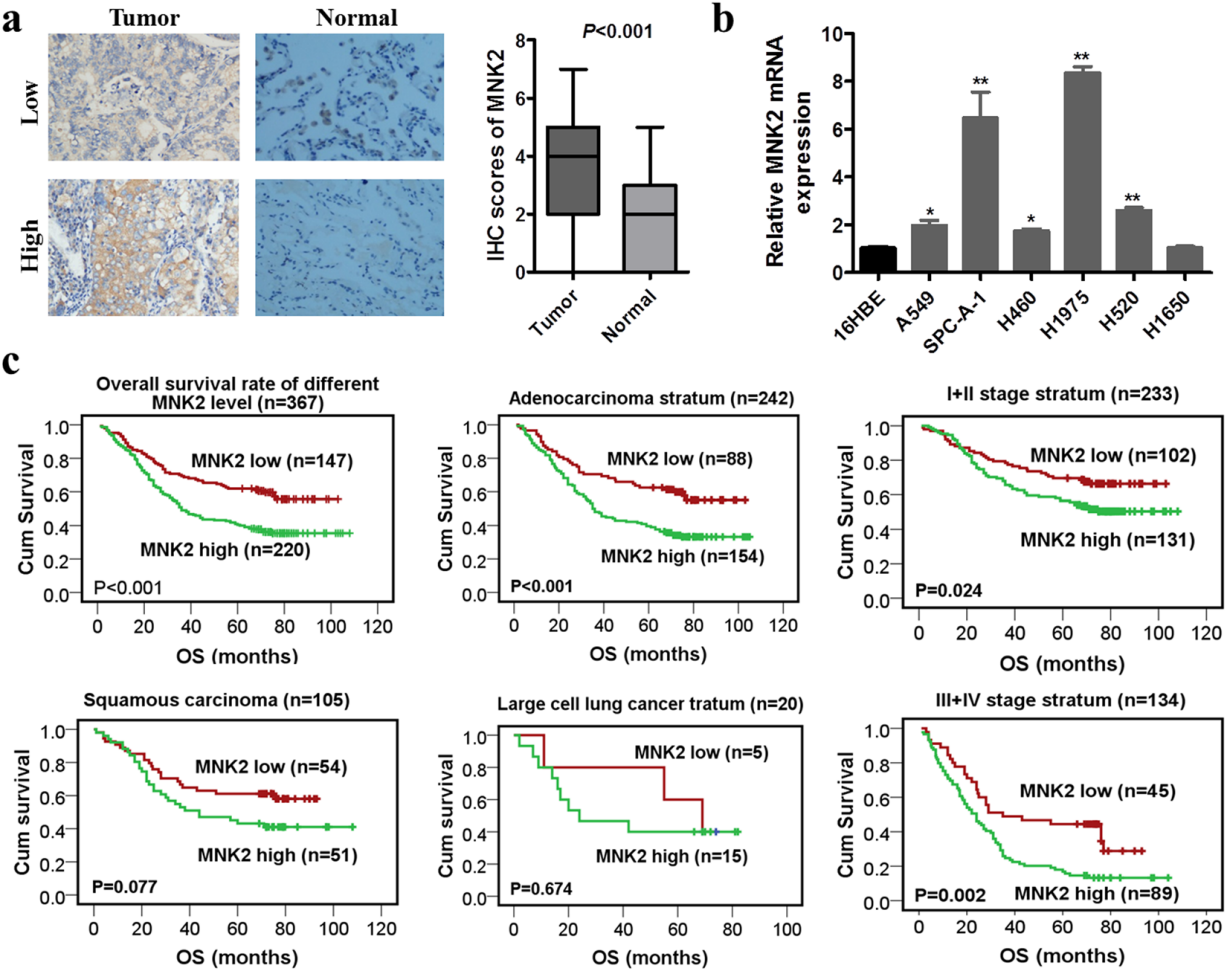
MNK2 was overexpressed in NSCLC and correlated with poorer prognosis. (**a**) MNK2 was highly expressed in NSCLC tissues compared with normal adjacent lung tissues (NATs) (**P < 0.01). Representative IHC staining of MNK2 in 2 pairs (low expression and high expression) of NSCLC cases (original magnification: ×200). (**b**) RT-PCR analysis showed that MNK2 was overexpressed in most NSCLC cell lines compared with normal bronchi epithelial cell line (16HBE) (*P < 0.05, **P < 0.01). (**c**) Kaplan-Meier analysis showed that the overall survival (OS) rates of NSCLC patients with MNK2 overexpression were lower, especially in adenocarcinoma and clinical stage III + IV (*P* < 0.01).

Expression of MNK2 was investigated by IHC with MNK2 monoclonal antibodies by using NSCLC tissue microarray that contained 367 NSCLC tissues and 117 normal lung tissues, compared with normal lung epithelial, higher expression of MNK2 in cancer tissues (*P* < 0.001; Fig. 1a). MNK2 was expressed in the cytoplasm. According to ROC curve analysis of MNK2 immunoreactivity, a score of MNK2 above the 3.5 cutoff value was defined as high expression, and high expression was found in 60.5% (222/367) of NSCLC tissues (Fig. 1a). RT-PCR was used to detect the levels of MNK2 mRNA in NSCLC and normal bronchi epithelial (16HBE) cell lines. Compared with 16HBE, MNK2 was elevated in H460, A549, SPC-A-1, H520, and H1975 cell lines between 1.67- and 8.05-fold (*P < 0.05; **P < 0.01; Fig. 1b).

### Clinical significance of MNK2 overexpression in NSCLC

In order to determine the correlation between MNK2 overexpression and NSCLC clinicopathological features, IHC data from 367 NSCLC cases were used. The results showed that MNK2 overexpression correlated with lymph node metastasis (*P* = 0.008; Table 1). Kaplan-Meier analysis showed that the OS rates of NSCLC patients were lower in those with high expression of MNK2 (*P* < 0.001); clinical stage and histology type were taken into consideration during stratification, higher MNK2 expression correlated with lower OS (*P* = 0.024) in stage I + II stratum, and higher MNK2 expression correlated with lower OS (*P* < 0.01) in stage III + IV and adenocarcinoma stratum (Fig. 1c). Multivariate Cox regression analysis demonstrated MNK2 overexpression as an independent prognostic factor for poor survival in patients with NSCLC (*P* = 0.003, Table 2). These findings suggested that MNK2 could play an important role in NSCLC proliferation and progression.

**Table 1:** Correlation between MNK2 expression and clinicopathological features in 367 primary NSCLCs.

Variable	MNK2 expression			
	Case	Low	High	*P* value
	N = 367	N = 147	N = 220	
Age (years)				
≥58.5	198	70	128	0.054
<58.5	169	77	92	
Gender				
Male	200	38	162	1
Female	167	109	58	
Smoking				
Yes	239	95	144	0.991
No	128	52	76	
Histology type				
ACC	242	88	154	0.056
SCC	105	54	51	
LCLC	20	5	15	
T stage				
1,	57	26	31	0.379
2,3,4	310	121	189	
LN metastasis				
No	191	89	102	0.008
Yes	176	58	118	
Clinical stage				
I, II	233	102	131	0.06
III, IV	134	45	89	

Abbreviations: NSCLC: Non-small cell lung cancer; ACC: adenocarcinoma; SCC: squamous cell cancer; LCLC: large cell lung cancer; LN: lymph node; T stage: T parameter of TNM classification.

**Table 2:** Effect of factors on overall survival in NSCLC patients in the univariate and multivariate cox regression model.

Factors	Univariate		Multivariate^a^	
	HR (95% CI)	*P* value	HR (95% CI)	*P* value
Age, ≥58.5 *vs* <58.5	1.408 (1.063–1.864)	0.017	1.312 (0.986–1.746)	0.063
Sex, female *vs* male	1.101 (0.804–1.510)	0.548	—	—
Smoking, Yes. *vs* No.	0.952 (0.714–1.269)	0.737	—	—
Histology type, ACC *vs* SCC	0.872 (0.649–1.173)	0.365	—	—
T stage, 1 *vs* 2,3,4	2.007 (1.277–3.154)	0.003	1.546 (0.974–2.454)	0.064
LN metastasis, Yes. *vs* No.	3.275 (2.437–4.401)	0	2.304 (1.513–3.508)	0
Clinical stage, I + II *vs* III + IV	2.876 (2.178–3.798)	0	1.475 (0.989–2.199)	0.057
MNK2, Low *vs* High	1.832 (1.358–2.472)	0	1.584 (1.167–2.151)	0.003

Abbreviations: NSCLC: Non-small cell lung cancer; ACC: adenocarcinoma; SCC: squamous cell cancer; LN: lymph node; T stage: T parameter of TNM classification. ^a^For the multivariate model, HR and P values were shown for the final set of stepwise selected variables only.

### MNK2 could promote cell proliferation, clonogenicity, and tumor growth

**Figure 2:**
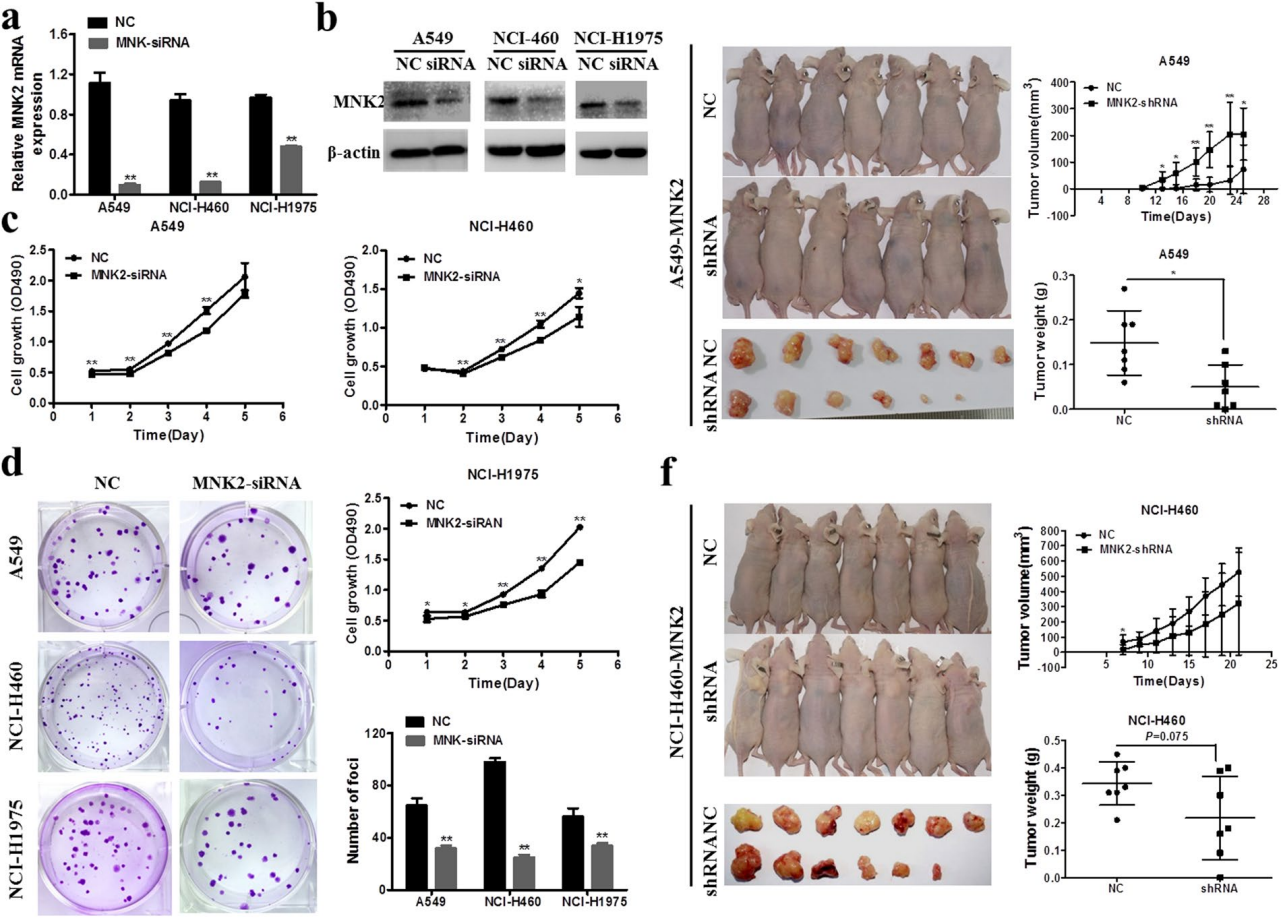
Silencing of MNK2 reduced cell and tumor growth. (**a,b**) Expression of MNK2 was silenced by siRNA. The mRNA and protein levels of MNK2 were detected by qRT-PCR and WB, respectively (**P < 0.01). siRNA-NC was used as a vector control. Images presented in this panel were cropped from different parts of the same gel, or from different gels. The full-length gels are presented in the Supplementary Fig. S1. (**c**) Cell proliferation rates of MNK2-silencing cells (siRNA-MNK2) and negative control cells were evaluated by CCK-8 assay. Results were expressed as mean ± SD of three independent experiments (*P < 0.05, **P < 0.01). (**d**) Foci formation assay was performed to compare the frequency of foci formation between siRNA-MNK2 and negative control cells. Results were expressed as mean ± SD of three independent experiments (**P < 0.01). (**e**) For A549 cell groups, tumor number and size of the shRNA-MNK2 group (n = 6) were less and smaller than those of the negative control group (n = 7). Detected tumor growth was significantly different (*P < 0.05, **P < 0.01). Tumor weights between each group were also compared (*P < 0.05). (**f**) For NCI-H460 cell groups, tumor number and size of the shRNA-MNK2 group (n = 6) were less and smaller than those of the negative control group (n = 7). However, no significant difference was detected for tumor growth (*P* > 0.05) except on the 7^th^ day (*P < 0.05). Tumor weights between the shRNA-MNK2 group and negative control group were also compared (*P* = 0.075).

To test the function of MNK2 in NSCLC, the A549, H460, and H1975 cell lines were silenced by RNA interference. Cells were transfected with siRNA targeting MNK2 (siRNA-MNK2) or the negative control (siRNA-NC). The messenger RNA and protein levels of MNK2 were detected by qRT-PCR and Western bolt, respectively (**P < 0.01; Fig. 2a,b). The tumorigenic effect of MNK2 was investigated by *in vitro* functional assays, including cell proliferation and foci formation assays. The results showed that the cell proliferation rate (Fig. 2c) and foci formation (Fig. 2d) were significantly decreased in siRNA-MNK2 cells (**P  < 0 0.01) when compared with those in negative control cells.

We next investigated whether MNK2 silencing could reduce tumor growth in nude mice by stable transduction of lentiviral vectors containing shRNA targeting MNK2 (shRNA-MNK2) or empty vector as negative control (shRNA-NC). The result (Fig. 2e) showed that A549 cells transduced with shRNA-MNK2 inhibited tumor growth significantly compared with those transduced with shRNA-NC when measured from the 10^th^ day (*P < 0.05, **P < 0.01); moreover, the mean tumor weight was lower in cells transduced with shRNA-MNK2 than in those transduced with shRNA-NC (*P < 0.05). For NCI-H460 cell groups (Fig. 2f), tumor masses were less (6/7; 85.7%) and smaller in the shRNA-MNK2 group than in the shRNA-NC group. Unexpectedly, no significant difference was detected for tumor growth (*P* > 0.05) except on the 7^th^ day (*P < 0.05), and for tumor weight (*P* = 0.075).

### MNK2 could inhibit cell apoptosis

**Figure 3:**
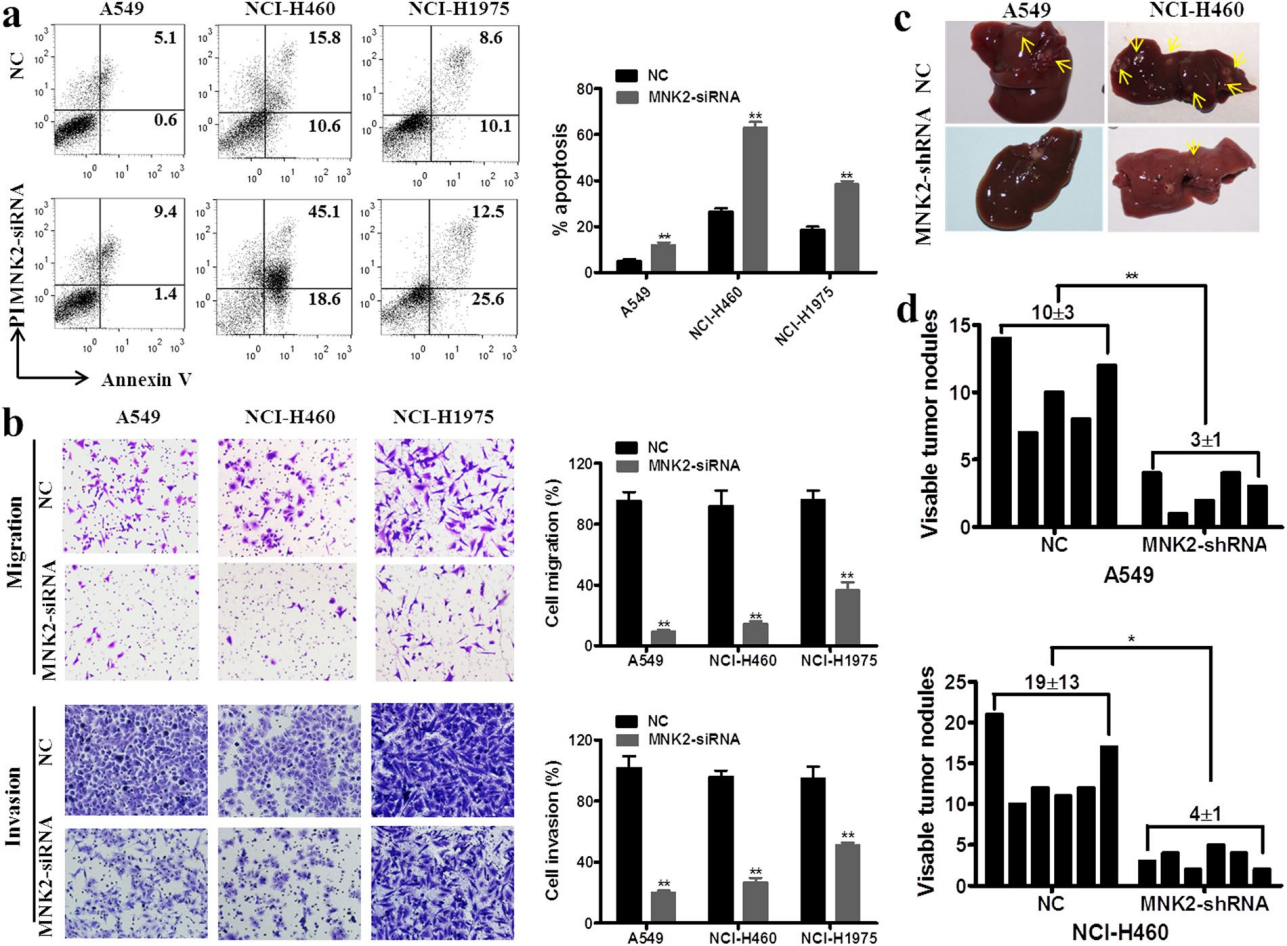
Silencing of MNK2 increased cell apoptosis, reduced cell migration, invasion and promote metastasis in NSCLC. (**a**) Annexin-V/propidium iodide (PI) assay was performed to cell apoptosis between cells treated with siRNA-MNK2 and negative control. The representative images are shown (Left). Results were summarized as mean ± SD of 3 independent experiments (**P < 0.01) (Right). (**b**) Cell migration and invasion were performed to compare cell motilities between cells treated with siRNA-MNK2 and negative control cells. The representative images are shown (Left). Results were summarized as mean ± SD of 3 independent experiments (**P < 0.01) (Right). (**c**,**d**) Representative images of metastatic nodules on the liver surface of tested mice (Left). Visible tumor nodules on the liver of tested mice were compared between A549 (**P < 0.01) and NCI-H460 (*P < 0.05) cell groups.

To determine whether MNK2 has an anti-apoptosis effect on NSCLC cells, we implemented a flow cytometry analysis. Annexin-V/PI dual staining in A549, NCI-H460 and NCI-H1975 cells, revealed that MNK2 knockdown with siRNA cause increase in cell apoptosis compared with which transfected with siRNA NC (**P < 0.01; Fig. 3a).

### MNK2 could promote cell migration, invasion and tumor metastasis

Clinical correlation analysis found that MNK2 overexpression was associated with NSCLC lymph node metastasis, suggesting that MNK2 could confer tumor cells with migration and invasion abilities; thus, its role in cell migration and invasion was investigated by both *in vitro* and *in vivo* assays. To test MNK2 effect on tumor cell migration, siRNA targeting MNK2 (siRNA-MNK2) was transfected into A549, NCI-H460, and NCI-H1975 cell lines. Cell migration assay showed that siRNA-MNK2 cells could significantly decrease cell migration and invasion compared with siRNA-NC cells (**P < 0.01; Fig. 3b).

To further determine the effect of MNK2 on tumor metastasis, *in vivo* metastasis assay was performed in nude mice; shRNA-MNK2 vectors or empty vectors as negative control (shRNA-NC) were stably transduced into A549 cells or NCI-H460 cells, which were then injected intravenously via tail vein. Four weeks later, tested mice were sacrificed and metastatic nodules formed on liver and lung surfaces were counted. In the hematogenous metastasis model, more nodules were observed on livers injected with shRNA-NC transduced A549 cells or NCI-H460 cells compared with those injected with shRNA-MNK2 cells (*P < 0.05, **P < 0.01, Fig. 3c,d). No apparent nodules were observed on the lung surface.

### MNK2 actives MNK2/4EBP1/eIF4E and ERK/MNK2/eIF4E pathway to promote NSCLC growth and metastasis

**Figure 4:**
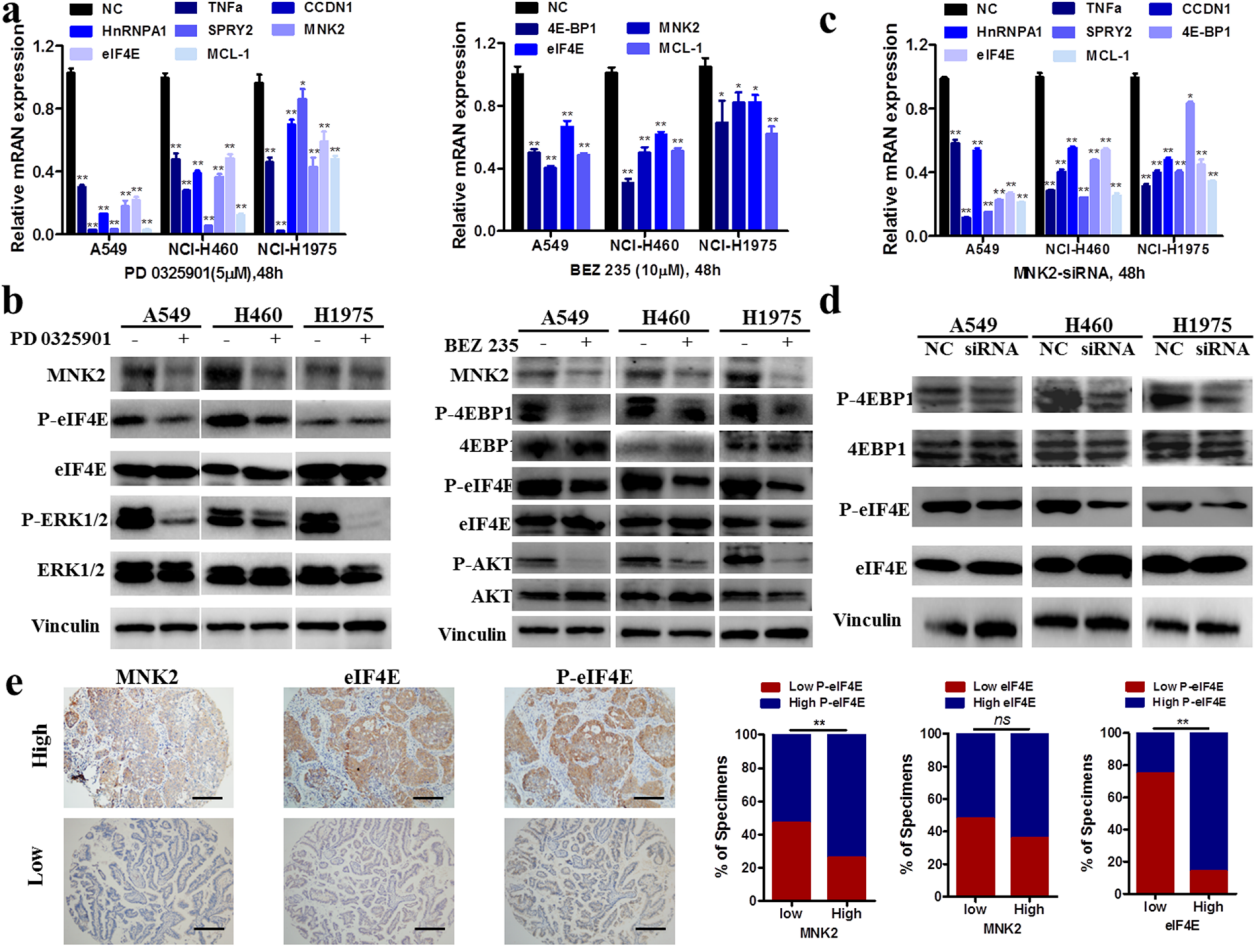
MNK2 actives AKT/4EBP1/eIF4E/MCL and ERK/eIF4E/MCL pathway. (**a**) A549, NCI-460 and NCI-1975 were treated with MEK inhibitor PD 0325901 and AKT inhibitor BEZ 235 (48h), respectively. The ERK and AKT downstream genes were detected by RT-PCR. Results were summarized as mean ± SD of 3 independent experiments (**P < 0.01). (**b**) Western bolt shown that inhibited MEK, both decrease MNK2 expression and eIF4E phosphorylation. Inhibited AKT, decreased MNK2 and eIF4E, 4EBP1 phosphorylation. (**c**) Silencing with siRNA-MNK2. RT-PCT of MNK2 response genes were down-regulated in the indicated cells. (**d**) Silencing MNK2 inactivated both 4EBP1 and eIF4E in the indicated cells. (**e**) p-eIF4E had a significantly positive correlation with MNK2 (**P < 0.01) in NSCLC tissues but not eIF4E. Representative IHC result was shown (original magnification: x40). Images presented in this panel were cropped from different parts of the same gel, or from different gels. The full-length gels are presented in the Supplementary Figs S2–S4.

TNFα, CCND1, HnRNPA1, SPRY2, eIF4E and MCL1 are MNK2 response genes^23–25^. To establish whether MNK2-regulated 4EBP1/eIF4E and ERK/eIF4E pathway promote tumor growth and metastasis in NSCLC, we used RT-PCR and western bolt analysis to measure mRNA and protein levels. A549, NCI-H460, and NCI-H1975were treated with PD 0325901 (5µM) or BEZ 235 (10µM) for 48h, and mRNA level of MNK2 response genes were detected by RT-PCR, the results showed that inhibit AKT or ERK both downregulate MNK2 and its response genes (**P < 0.01, Fig. 4a). In order to test whether MNK2 could phosphorylate the AKT or ERK pathway, western bolt analysis showed that inhibiting ERK downregulate MNK2 and decrease phosphorylation of eIF4E, inhibiting AKT both downregulate MNK2 and inactive 4EBP1 and eIF4E (Fig. 4b). Silencing MNK2, mRNA level of MNK2 response genes were decreased compared with the negative control group in A549, NCI-H460, and NCI-H1975 cell lines (**P < 0.01, Fig. 4c). Western bolt analysis revealed that 4EBP1 and eIF4E phosphorylation inactive when MNK2 was silenced by siRNA (Fig. 4d). Furthermore, we detected eIF4E and p-eIF4E in 174 matched cases of NSCLC tissues; the results showed that there was a significantly positive correlation between MNK2 and p-eIF4E (**P < 0.01, Fig. 4e) but not eIF4E (*P* > 0.05; Fig. 4e).

## Discussion

MNK2 has high basal activity because of the C termini, a striking feature of MNK2 that determines its ability to bind phosphorylated ERK^5^. Endogenous MNK1/2 kinases can enhance tumorigenesis associated with loss of Pten and are essential for eIF4E phosphorylation in transformed Pten-null cells *in vivo* ^17^. MNK2 inhibition results in down-regulation of eIF4E phosphorylation that correlates with AKT/mTOR pathway stability and up-regulation^13^. In the present study, MNK2 overexpression was detected in 60.5% of NSCLC tissues, which was significantly correlated with lymph node metastasis (*P* < 0.01) and poor overall survival rate (*P* < 0.001). Furthermore, multivariate Cox regression analysis revealed MNK2 up-regulation as an independent prognostic factor for poor survival in patients with NSCLC.

One important question raised was the means by which MNK2 could function in NSCLC. It was reported that eIF4E is highly phosphorylated and overexpressed in some solid tumors, including NSCLC, prostate cancer, breast cancer, penile squamous cell carcinoma, where eIF4E phosphorylation correlates with poor survival and tumor progression^7–9, 11^. MNK2 mainly contributes to the basal and constitutive phosphorylation of eIF4E, as basal eIF4E phosphorylation levels were decreased in MNK2−/− mice^5^. It was indicated in NSCLC that eIF4E is phosphorylated by MNKs, where overexpression of phosphorylated eIF4E predicts poor survival and correlates with tumor progression^7^. In this study, we silenced MNK2 with siRNA *in vitro*, which showed that MNK2 could promote NSCLC cells proliferation, migration and invasion. Furthermore, we stably transduced lentiviral vectors containing shRNA targeting MNK2 in cancer cells; the results demonstrated that MNK2 could promote tumor growth and metastasis *in vivo*. Clinical correlation analysis also suggested that MNK2 overexpression was correlated with lymph node metastasis. Interestingly, MNK2 elevation particularly correlated with clinical stage III + IV and adenocarcinoma, indicating that MNK2 might play a role in lately progress of lung adenocarcinoma (*P* < 0.01).

Another question raised was the mechanism by which MNK2 could promote tumor proliferation and metastasis. MNK1/2 phosphorylation of eIF4E on serine 209 involves anti-apoptotic activity enhancement, and eIF4E’s oncogenic action promotes tumorigenesis, tumor development, and cell transformation^9, 10, 12, 13, 15, 16^. The correlation of p-eIF4E with disease grade, disease early-onset, and poor prognosis in pancreatic ductal adenocarcinoma depends on MNK2 phosphorylation of eIF4E. MNK1/2 depletion resulted in the proportionate decrease of serine 209-phosphorylated eIF4E protein that matched the decrease achieved by the depletion of eIF4E itself by using eIF4E-specific siRNA^26^. In prostate cancer cells, the relative activity of AKT/mTOR and MNK/eIF4E pathways are under a controlled balance; mTOR and MNK concomitant inhibition had higher suppression of prostate cancer cell cycle progression and cell proliferation than inhibition of only one of them via phosphorylated eIF4E^9^. Recent report showed that combination inhibited by MNK inhibitor CGP57380 and mTOR inhibitor RAD001 exert synergistic antitumor efficacy in NSCLC, demonstrated targeting both mTOR and MNK/eIF4E signaling pathways can enhance the treatment^27^. In the present study, inhibiting AKT or ERK by inhibitor both down-regulate MNK2 and reduced eIF4E phosphorylation. And silencing of MNK2 both reduced eIF4E and 4EBP1 phosphorylation. In NSCLC tissues, there were positive correlation of MNK2 and p-eIF4E. Thus, indicating that MNK2 promote NSCLC tumor growth and progression via 4EBP1/eIF4E and ERK/eIF4E pathway.

Recently, it was reported that cercosporamide is a potent MNK inhibitor, particularly for MNK2. In acute myeloid leukemia cells, MNK2 inhibited by cercosporamide could decrease phosphorylation of eIF4E on serine 209, and such an inhibitory effect correlated with suppression of leukemic cell proliferation *in vitro*. The phosphorylation of eIF4E on serine 209 was reduced in xenografted tumors treated with cercosporamide; the abrogation of eIF4E phosphorylation by cercosporamide could also be enhanced with rapamycin^28^. In addition, recent report shown that BAY 1143269, a novel MNK1 inhibitor, has a strongly efficacy in monotherapy in NSCLC both *in vitro* and *in vivo*, by regulating cell cycle regulation, apoptosis, immune response and EMT^29^. There is another MNK1 inhibitor, CG957380, abrogating rapalogs-induced EIF4E phosphorylation and AKT activation and increase apoptosis to inhibit growth of NSCLC *in vitro* and *in vivo* ^27^. In pancreatic ductal adenocarcinoma, pharmacological inhibition of MNK activity synergistically enhanced the cytostatic effect of gemcitabine by promoting apoptosis. eIF4E phosphorylation is a general, long-lasting feedback response of pancreatic ductal adenocarcinoma cells to therapeutic treatments; inhibition of eIF4E phosphorylation enhances the cytostatic effect of therapeutic drugs, as demonstrated that MNK2 was required for resistance of pancreatic ductal adenocarcinoma cells to gemcitabine through the MNK/eIF4E pathway^30^. In our study, we inhibited ERK, the result showed that MNK2 response gene CCND1 was down-regulated in mRNA level. In order to test whether MNK2 has an apoptosis effect on NSCLC, apoptosis assay was used to demonstrate that silencing MNK2 increase cell apoptosis (*P* < 0.01). We demonstrated that silencing MNK2 can both inhibited NSCLC proliferation and metastasis. According to these results, we believe that up-regulation of MNK2 promotes tumor proliferation and metastasis via phosphorylated eIF4E in NSCLC, leading to poorer prognosis, later clinical stage, and higher chance of lymph node metastasis. More importantly, MNK2 depletion is essential for normal growth^5^. Inhibitors that target the frequently overexpressed oncogene MNK2 might elucidate the effective treatment of NSCLC with fewer side effects in the future.

Overall, MNK2 promotes tumor proliferation, migration, invasion and metastasis in NSCLC *in vitro* and *in vivo* via 4EBP1/eIF4E and ERK/eIF4E pathway. MNK2 has therapeutic potential against NSCLC progress. Further experiments are needed to elucidate the mechanism of MNK2, interaction of MNK2, and chemotherapeutics for NSCLC.

## Materials and Methods

### Tissue specimen and tissue microarray (TMA) construction

For immunohistochemical (IHC) staining, 367 cases of formalin-fixed paraffin-embedded NSCLC tissues and 117 cases of normal adjacent lung tissues (NATs, >2 cm from tumor tissues) were obtained from Sun Yat-sen University Cancer Center and The First Affiliated Hospital of Guangzhou Medical University (Guangzhou, China) between January 2007 and December 2010 with informed consent and agreement, and available clinicopathologic information. A pathologist (Q.N.W.) reviewed slides from all blocks, selecting representative areas of NSCLC tissue to be duplicated by a MiniCore Tissue Arrayer with a 1-mm needle. Two pathologists (Q.N.W. and Y.Z.) independently confirmed the diagnosis and histological grade of each case based on World Health Organization classification^31^. The clinical stage was classified according to the American Joint Committee on Cancer (AJCC) and tumor-lymph node-metastasis (TMN) classification system^32^. The study was performed according to the Chinese National Ethical Guidelines (Code for Proper Secondary Use of Human Tissues, Chinese Federation of Medical Scientific Societies) and approved by the Research Ethics Committee of Guangzhou Medical University.

### Cell lines and cell culture

NSCLC cell lines (A549, NCI-H460, SPC-A-1, NCI-H1650, NCI-H520, NCI-H1975) and normal bronchi epithelial cell line (16HBE) were kindly provided by professor J.H.J. (State Key Laboratory of Respiratory Disease, Guangzhou). They were incubated in RPMI-1640 medium supplemented with 10% fetal bovine serum (Gibco, Grand Island, New York), 100 units/ml of penicillin, and 100 µg/ml of streptomycin. All cells were maintained in a humidified incubator (37 °C, 5% CO_2_).

### RNA isolation, cDNA synthesis, and real-time polymerase chain reaction (RT-PCR)

Total RNA was isolated using Trizol reagent (Invitrogen). cDNA was synthesized using MultiScribe Reverse Transcriptase (ABI, Calsbad, CA). RT-PCR was carried out using SYBR Green QPCR Master Mix (Stratagene, USA) with the MX3000P Real- Time PCR system (Stratagene, Santa Clara, California). All primers for amplification were purchased from GenePharma (Shanghai, China). GAPDH gene was amplified as an internal control. The relative expression was calculated using the relative quantification equation, RQ = 2^−ΔΔCT^ ^24^.

### Reagents and antibodies

Antibodies used for immunoblots were MNK2 (1:300 dilution; Abcam), eIF4E (1:5000 dilution; Abcam), P-eIF4E (1:500 dilution; Abcam), ERK1/2 (1:1000 dilution; Abcam), P-ERK1/2 (1:1000 dilution; Abcam), 4EBP1 (1:1000 dilution; Cell Signaling Technology), P-4EBP1 (1:1000 dilution; Cell Signaling Technology), AKT (1:1000 dilution; Affinity), P-AKT (1:1000 dilution; Affinity), β-actin (1:5000 dilution; Abcam), and Vinculin (1:5000 dilution; Abcam). AnnexinV/PI (KeyGEN). MER Inhibitor PD 0325901 and AKT inhibitor BEZ 235 were purchased from Selleck Chemicals.

### Western blotting

Quantified protein lysates were resolved on SDS-PAGE gel, transferred onto PVDF membrane (Millipore, Billerica, MA), and immunoblotted with anti-human antibodies. Blots were visualized with an enhanced chemiluminescence kit (Thermo Pierce ECL Western Blotting Substrate) with Bio-Rad ChemiDoc MP (California, USA). Images were analyzed by Bio-Rad Image Lab software.

### Transfection with siRNA (RNA interference)

Small interfering RNA (siRNA) duplexes specific for MNK2 or the negative control siRNA were purchased from Genepharma (Shanghai, China). siRNA (60 nM) against MNK2 (siRNA-MNK2) or the negative control (siRNA-NC) was transfected into cells in 6-well plates using Lipofectamine 2000 reagent (Invitrogen, Carlsbad, CA) according to the manufacturer’s instructions. The cells transfected with siRNA-NC were used as negative controls. At 48 hours and 72 hours after transfection, the effects of gene silencing were measured via RT-PCR and western blot analysis.

### Lentiviral transduction

Lentiviral constructs harboring shRNA targeting MNK2 (Genepharma, China) were packaged using the ViraPower Mix (Invitrogen, Carlsbad, CA) in 293A cells. Vectors expressing shRNA targeting MNK2 (shRNA-MNK2) were used to stably transduce NSCLC cells (A549 and NCI-H460) in establishing stable MNK2-silencing cells. Empty vector-transduced cells were established as negative controls (shRNA-NC).

### Cell proliferation assay

For cell proliferation assay, cells were transfected with siRNA specific for MNK2 (siRNA-MNK2) or negative control (siRNA-NC) Genepharma, shanghai, China) 48 hours before the experiment, and cells were then seeded onto a 96-well plate at 2 × 10^3^ cells per well. Cell proliferation rates were evaluated by the CCK-8 kit assay (Dojindo, Japan) according to the manufacturer’s instructions. Three independent assays were performed.

### Foci formation assay

For foci formation assay, cells were transfected with siRNA specific for MNK2 (siRNA-MNK2) or negative control (siRNA-NC) (Genepharma, China) 48 hours before the experiment; 500 cells were plated in 6-well plates for 10 days. Surviving colonies were then visualized by 1% crystal violet staining, and colonies with no less than 50 cells/colony were counted. Three independent experiments were performed.

### Transwell migration and invasion assays

Cells suspended in serum-free RPMI-1640 were seeded into chambers with an 8-mm microporous filter (Becton Dickson Labware, Bedford, MA) to observe migration. Cells were also suspended in RPMI-1640 with free fetal bovine serum into chambers with matrix gel (Corning, New York, USA) to observe invasion. After 30 hours, cells were fixed, stained, and counted. Three independent experiments were performed.

### Xenograft formation assay

Cells were classified as stable MNK2-inhibition (shRNA-MNK2) or negative control (shRNA-NC). A549 cells (shRNA-MNK2 or shRNA-NC) (1 × 10^6^ cells per mice) or NCI-H460 cells (shRNA-MNK2 or shRNA-NC) (3 × 10^6^ cells per mice) were injected subcutaneously into the dorsal right flank of 5- to 6-week-old BALB/c nude mice (n = 7 per group). For A549 cell groups, tumor diameter and width were measured every 3-4 days from the 10^th^ day after inoculation until the 28^th^ day. For NCI-H460 cell groups, tumor sizes were measured every 3 days from the 7^th^ day after inoculation until the 28^th^ day. The tumor volume was calculated by the formula, V = 0.5 × L × W^2^. The Animal Experimentation Ethics Committee of Guangzhou Medical University approved experiments on animals.

### *In vivo* metastasis assay

For *in vivo* metastasis assays, 1 × 10^6^ stably transduced cells (shRNA-MNK2 or shRNA-NC) were injected intravenously through the tail vein into 4- to 5-week-old BALB/c nude mice (6 mice for NCI-H460 group and 5 mice for A549 group). The mice were sacrificed 4 weeks later, and the intrahepatic metastatic nodules were carefully examined and counted. The Animal Experimentation Ethics Committee of Guangzhou Medical University approved experiments on animals. We confirmed that all experiments were performed in accordance with relevant guidelines and regulations.

### IHC staining and selection of cutoff score

IHC staining was performed using the standard streptavidin-biotin-peroxidase complex method. Paraffin NSCLC tissues and normal lung tissues were cut into 5-µm sections that were deparaffinized and incubated with MNK2, eIF4E, and p-eIF4E polyclonal antibodies (1:200 dilution, Abcam) overnight at 4 °C. Tissue sections were then incubated with biotinylated goat anti-rabbit immunoglobulin at a concentration of 1:75 at 37 °C for 30 min. Finally, the sections were developed with diaminobenzidine (DAB). Three pathologists (Q.N.W., S.Y.X., and Y.Z.) independently assessed the slides without knowledge of clinicopathologic data. Variant cases were reviewed and discussed until a consensus was obtained. Five areas were selected at random and scored. The percentage of tumor cells with positive staining was determined at high magnification (x200). The scores for percentage of positive cells (<5%, 0; 5–25%, 1; 25–50%, 2; 50–75%, 3; >75%, 4) and the intensity of positive staining (negative, 0; weak, 1; moderate, 2; or strong, 3) were determined as previously described^33, 34^. Receiver operating characteristic (ROC) curve analysis was performed to determine the cutoff score for a “high expression” designation with the 0, 1-criterion implemented in SPSS statistical software.

### Statistical analysis

Data were expressed as mean ± SD. *t*-tests were used to analyze the significance of differences. The correlation between MNK2 expression and clinicopathological features of NSCLC patients was analyzed by the χ^2^ test. Survival curves were generated according to the Kaplan-Meier method, and statistical analysis was performed using the log-rank test. The Cox proportional hazards regression model was used to identify independent prognostic factors. Pearson’s correlation was applied to compare the expression between MNK2 and eIF4E or P-eIF4E. All statistical analyses were performed using statistical software (SPSS 19.0 for Windows; SPSS Inc., Chicago, IL). Differences were considered significant when *P* < 0.05.

### Data Availability

The datasets generated during and/or analysed during the current study are available from the corresponding author on reasonable request.

## Electronic supplementary material

Supplementary Information
