# Supplementary material for: MAP kinase-interacting serine/threonine kinase 2 promotes proliferation, metastasis, and predicts poor prognosis in non-small cell lung cancer
Source: Sci Rep. 2017 Sep 6;7:10612. doi: 10.1038/s41598-017-10397-9 (PMC5587555; doi:10.1038/s41598-017-10397-9)

MAP kinase-interacting serine/threonine kinase 2 promotes proliferation, metastasis, and predicts poor prognosis in non-small cell lung cancer

Zhihua Guo[1,2,3,†]; Guilin Peng[1,2,3,†]; Ermao Li[4]; Shaoyan Xi[5]; Yu Zhang[5];Yong Li[5]; Xiaodong Lin[6]; Guangqiu Li[6]; Qinian Wu[6,*]and Jianxing He[1,2,3,*]

Address to correspondence to:

Qinian Wu, MD, PhD. Department of Pathology, The First Affiliated Hospital of Guangzhou Medical University, No. 151, Yanjiang Rd, Guangzhou 510120, China. wuqinian1028@163.com; 86 20 83062546.

Jianxing He, MD, PhD. Department of Thoracic Surgery, The First Affiliated Hospital of Guangzhou Medical University, No. 151, Yanjiang Rd, Guangzhou 510120, China. drjianxing.he@gmail.com; 86 20 83062822.

Supplementary Figure S1. Expression of MNK2 was silenced by siRNA. The protein levels of MNK2 was detected by WB (**P < 0.01). siRNA-NC was used as a vector control. Images presented in this panel were cropped from different parts of the same gel, or from different gels. Full-length gels are shown.

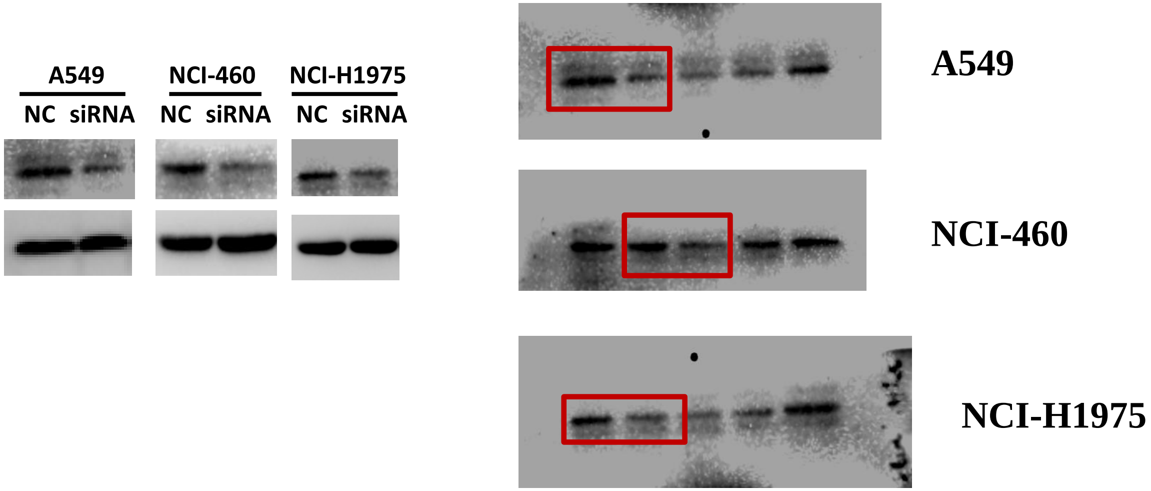

Supplementary Figure S2. Western bolt shown that inhibited MEK, both decrease Mnk2 expression and eIF4E phosphorylation. Inhibited AKT, decreased MNK2 and eIF4E, 4EBP1 phosphorylation. Images presented in this panel were cropped from different parts of the same gel, or from different gels. Full-length gels are shown.

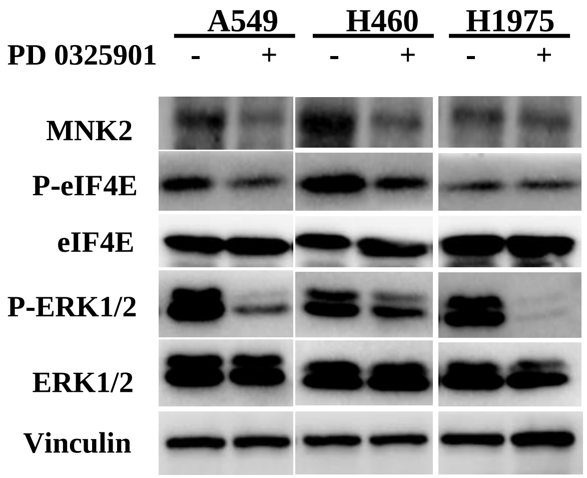

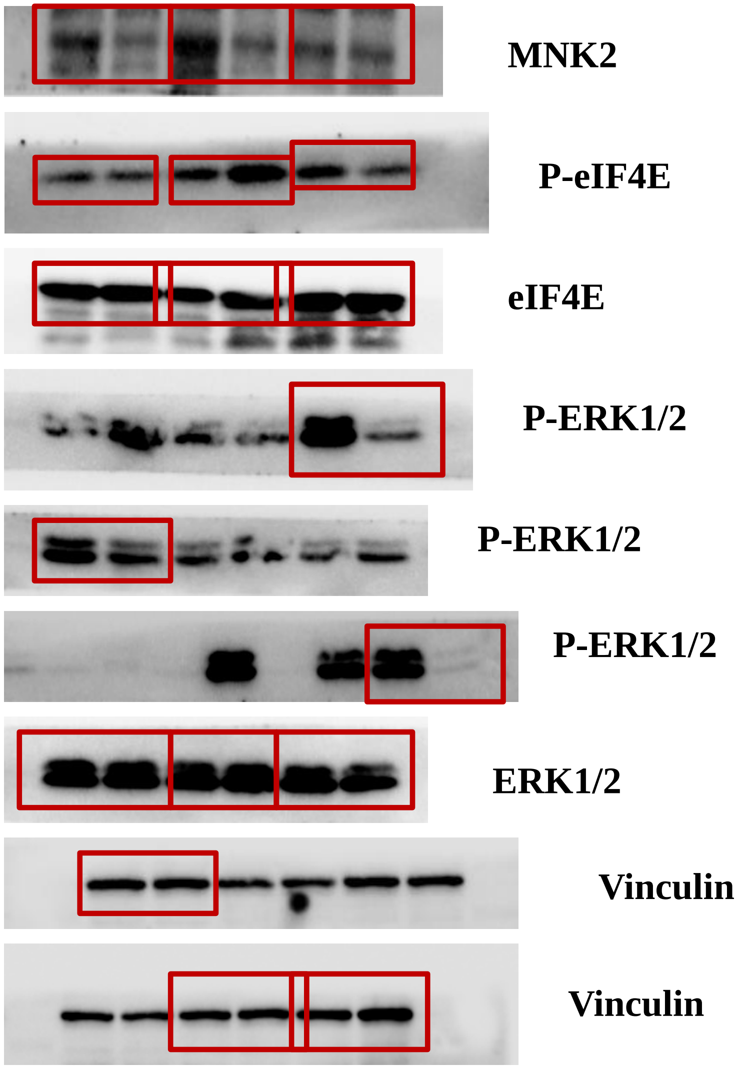

Supplementary Figure S3. Western bolt shown that inhibited MEK, both decrease Mnk2 expression and eIF4E phosphorylation. Inhibited AKT, decreased MNK2 and eIF4E, 4EBP1 phosphorylation. Images presented in this panel were cropped from different parts of the same gel, or from different gels. Full-length gels are shown.

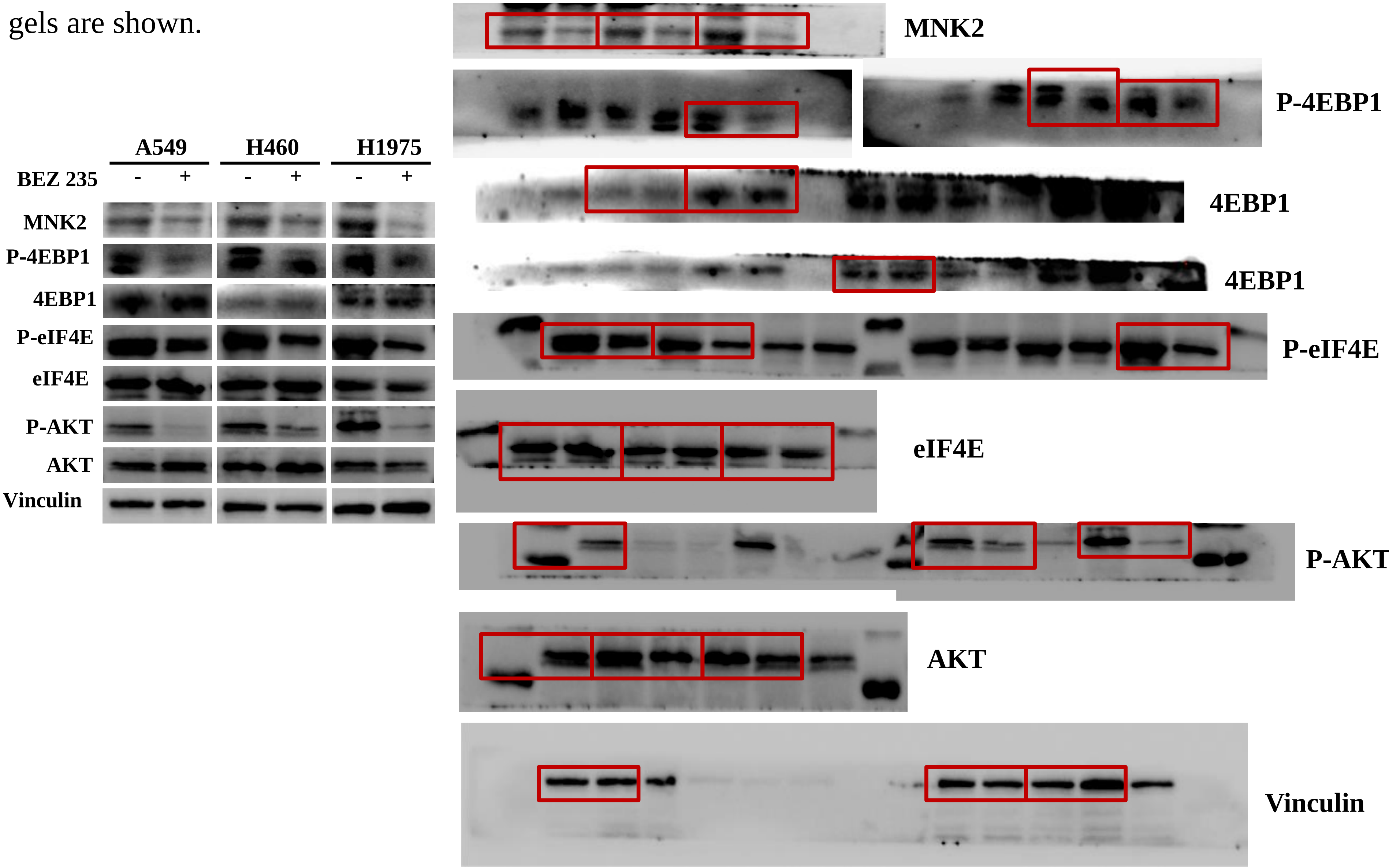

Supplementary Figure S4. Silencing Mnk2 inactivated both 4EBP1 and eIF4E in the indicated cells. Images presented in this panel were cropped from different parts of the same gel, or from different gels. Full-length gels are shown.

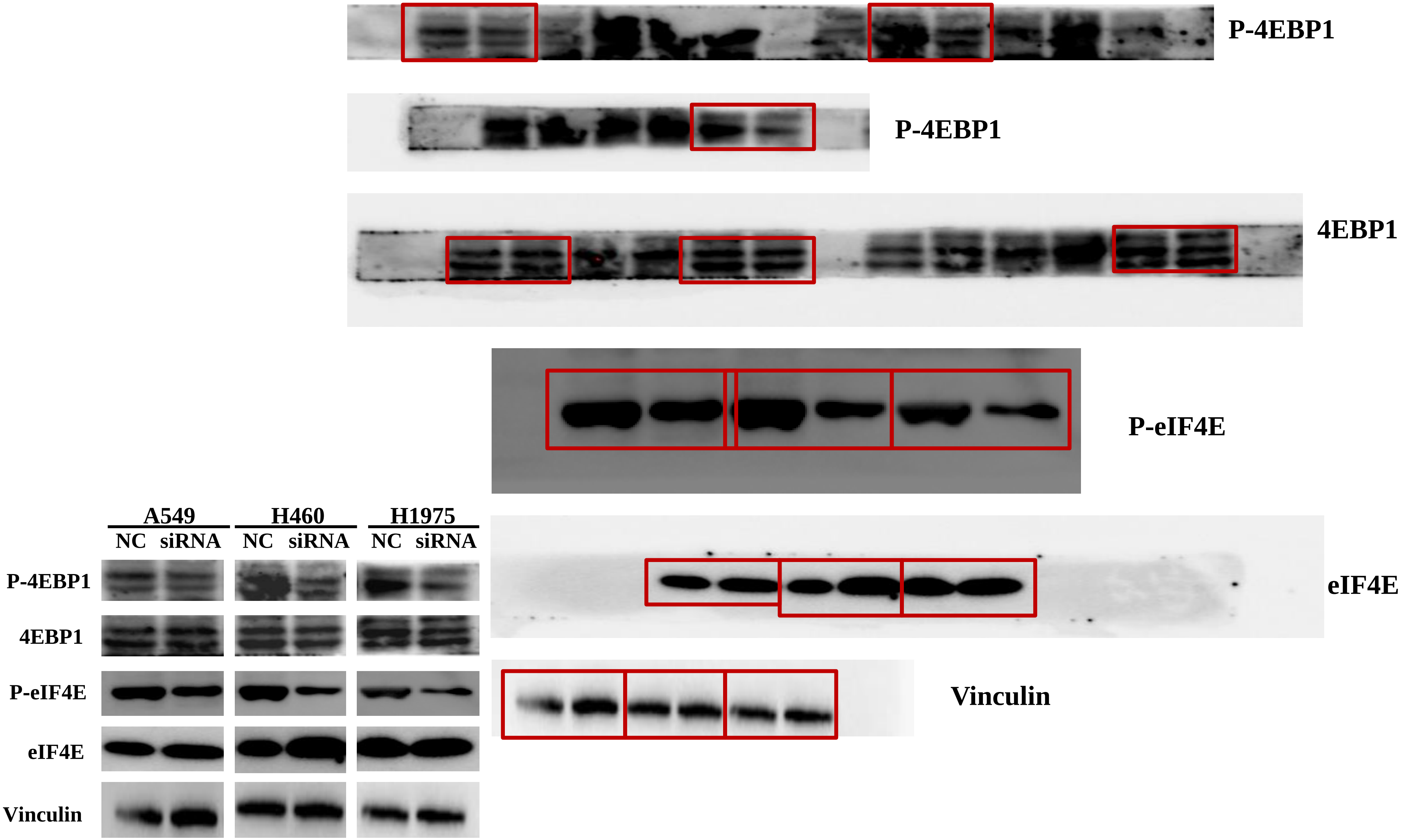

Supplement: Supplementary file 1 — Supplementary Information [file 41598_2017_10397_MOESM1_ESM.pdf]
